# Roads constrain movement across behavioural processes in a partially migratory ungulate

**DOI:** 10.1186/s40462-021-00292-4

**Published:** 2021-11-13

**Authors:** Gioele Passoni, Tim Coulson, Nathan Ranc, Andrea Corradini, A. J. Mark Hewison, Simone Ciuti, Benedikt Gehr, Marco Heurich, Falko Brieger, Robin Sandfort, Atle Mysterud, Niko Balkenhol, Francesca Cagnacci

**Affiliations:** 1 Department of Zoology, University of Oxford, Zoology Research and Administration Building, 11a Mansfield Rd, Oxford, OX1 3SZ UK; 2 Department of Biodiversity and Molecular Ecology, Research and Innovation Centre (CRI), Fondazione Edmund Mach, Via Edmund Mach 1, 38010 San Michele all’Adige, TN Italy; 3 Center for Integrated Spatial Research, Environmental Studies Department, University of California, Santa Cruz, 95064 USA; 4 Department of Civil, Environmental and Mechanical Engineering (DICAM), University of Trento, via Mesiano 77, 38123 Trento, TN Italy; 5 Stelvio National Park, Via De Simoni 42, 23032 Bormio, SO Italy; 6 INRAE, CEFS, Université de Toulouse, 31326 Castanet-Tolosan, France; 7 LTSER ZA Pyrénées Garonne, 31320 Auzeville Tolosane, France; 8 Laboratory of Wildlife Ecology and Behaviour, University College Dublin, Belfield, D4 Ireland; 9 Department of Evolutionary Biology and Environmental Studies, University of Zurich, Winterthurerstrasse 190, 8057 Zurich, Switzerland; 10 Department of Visitor Management and National Park Monitoring, Bavarian Forest National Park, Freyunger Straße 2, 94481 Grafenau, Germany; 11 Faculty of Environment and Natural Resources, Chair of Wildlife Ecology and Management, University of Freiburg, Tennenbacher Straße 4, 79106 Freiburg, Germany; 12 Institute for Forest and Wildlife Management, Inland Norway University of Applied Science, 2480 Koppang, Norway; 13 Wildlife Institute, Forest Research Institute Baden-Wuerttemberg, Wonnhaldestraße 4, 79100 Freiburg, Germany; 14 Department of Integrative Biology and Biodiversity Research, Institute of Wildlife Biology and Game Management, University of Natural Resources and Life Sciences Vienna, Gregor‐Mendel Straße 33, 1180 Vienna, Austria; 15 Centre for Ecological and Evolutionary Synthesis (CEES), Department of Biosciences, University of Oslo, Blindern, P.O. Box 1066, 0316 Oslo, Norway; 16 Wildlife Sciences, Faculty of Forest Sciences and Forest Ecology, University of Goettingen, Buesgenweg 3, 37077 Goettingen, Germany

**Keywords:** Ungulates, Roe deer, *Capreolus capreolus*, Migration, Dispersal, Roads, Habitat selection, Step selection analysis, Connectivity

## Abstract

**Background:**

Human disturbance alters animal movement globally and infrastructure, such as roads, can act as physical barriers that impact behaviour across multiple spatial scales. In ungulates, roads can particularly hamper key ecological processes such as dispersal and migration, which ensure functional connectivity among populations, and may be particularly important for population performance in highly human-dominated landscapes. The impact of roads on some aspects of ungulate behaviour has already been studied. However, potential differences in response to roads during migration, dispersal and home range movements have never been evaluated. Addressing these issues is particularly important to assess the resistance of European landscapes to the range of wildlife movement processes, and to evaluate how animals adjust to anthropogenic constraints.

**Methods:**

We analysed 95 GPS trajectories from 6 populations of European roe deer (*Capreolus capreolus*) across the Alps and central Europe. We investigated how roe deer movements were affected by landscape characteristics, including roads, and we evaluated potential differences in road avoidance among resident, migratory and dispersing animals (hereafter, movement modes). First, using Net Squared Displacement and a spatio-temporal clustering algorithm, we classified individuals as residents, migrants or dispersers. We then identified the start and end dates of the migration and dispersal trajectories, and retained only the GPS locations that fell between those dates (i.e., during transience). Finally, we used the resulting trajectories to perform an integrated step selection analysis.

**Results:**

We found that roe deer moved through more forested areas during the day and visited less forested areas at night. They also minimised elevation gains and losses along their movement trajectories. Road crossings were strongly avoided at all times of day, but when they occurred, they were more likely to occur during longer steps and in more forested areas. Road avoidance did not vary among movement modes and, during dispersal and migration, it remained high and consistent with that expressed during home range movements.

**Conclusions:**

Roads can represent a major constraint to movement across modes and populations, potentially limiting functional connectivity at multiple ecological scales. In particular, they can affect migrating individuals that track seasonal resources, and dispersing animals searching for novel ranges.

**Supplementary Information:**

The online version contains supplementary material available at 10.1186/s40462-021-00292-4.

## Background

Human disturbance negatively impacts wildlife and limits animal movement globally [69]. Infrastructure, such as roads, can reduce and fragment available habitat, increase wildlife mortality due to collisions with vehicles, and act as physical barriers that can impact behaviour across a range of spatial scales [27, 29]. In recent decades, the discipline of road ecology has developed to address these issues, focusing on various animal taxa and regions [41, 68]. In ungulates, roads can particularly hamper movement processes such as dispersal and migration (hereafter called “long range movements”), which are typically associated with long distance movements and key ecological processes [5].

Seasonal migration allows individuals to exploit spatial variation in resource availability, potentially reducing intra-specific competition and, in some cases, predation risk [35, 50]. Dispersal occurs when individuals leave their natal (or breeding) area to settle in their first (or a new) breeding range, impacting population genetic structure and metapopulation dynamics [9, 17, 66]. Long range movements ensure functional connectivity among populations, and may be particularly important for population performance in highly human dominated and mountainous areas where anthropogenic or topographic barriers may separate suitable habitat (e.g., roads, mountain ridges) [9].

In ungulates, movement and habitat selection are mainly shaped by landscape features such as topography and forest cover [19], seasonal dynamics like vegetation phenology [2], and human disturbance [56]. Some of these aspects, in particular the impact of roads on resident, migratory or dispersing animals, have already been investigated in a number of systems. For example, roads significantly reduced long-distance movements in reindeer (*Rangifer tarandus*) in Norway [6], and limited gene flow among different populations of roe deer in Switzerland [39]. Mule deer (*Odocoileus hemionus*) were also shown to avoid roads and move faster when migrating across roads [56].

Despite this body of knowledge, potential differences in response to roads during different movement modes, in particular residency, dispersal and migration as observed in partially migratory populations, have never been evaluated. Since habitat selection along these movement modes can differ [38], animals may show different responses to semi-permeable barriers such as roads. In resident individuals, roads can alter space use within the home range [8, 19] and increase home range size and mortality risk [36, 46]. Indeed, mortality risk was shown to be higher for roe deer (*Capreolus capreolus*) moving in unfamiliar areas on the outskirts of their home range [32]. By shaping space use [52, 53], site familiarity could therefore lead to a relatively strong road avoidance in resident individuals.

Long distance movements can also be costly in human-dominated environments, as they increase the likelihood of crossing roads [59]. Moving into new areas carries costs, and venturing out of an established home range can force individuals through areas with higher road densities. For example, Benoit et al. [5] showed that dispersing roe deer spent more energy than resident individuals, especially when moving through fragmented areas with high road densities. Additionally, site familiarity during long range movements can vary. Migration is a recurrent behaviour that generally occurs annually and can be culturally transmitted [37]. While migrating, animals may therefore have some familiarity with the surrounding landscape [58], and may be able to avoid roads more effectively. On the other hand, dispersal mostly occurs through unfamiliar landscapes [24]. As such, knowledge of the spatial distribution of roads and associated sources of risk is likely lower or absent. Addressing these issues is particularly important to assess the resistance of human-dominated landscapes to the range of different wildlife movements, and to evaluate how animals adjust their behaviour to anthropogenic constraints.

In this study, we focused on six populations of European roe deer across the Alps and central Europe, with two main objectives: (i) to investigate how roe deer movements are affected by roads and other landscape characteristics; and (ii) to identify potential differences in road avoidance among resident, migratory and dispersing animals. The European roe deer is a widespread ungulate in Europe that lives in habitats as diverse as boreal forests and Mediterranean shrublands [42]. They are behaviourally flexible, showing a wide variety of movement modes, from sedentary behaviour to migration, as well as several tactics of natal dispersal [26]. In the populations considered in this study, the roe deer is a partial and facultative migrator, with only part of the population migrating each year, generally following the elevation gradient [13, 50]. In turn, roe deer dispersal is a relatively conserved behaviour across populations [30].

To investigate movement-specific habitat selection and test for differences in road avoidance among different movement modes, we performed an integrated step selection analysis (iSSA, [4]). We expected roe deer movements to be affected by resource availability, landscape topography and risk avoidance. With regards to resources, we predicted that roe deer should select areas with higher NDVI values (Normalised Difference Vegetation Index—i.e., a proxy for the availability of food). Roe deer should also minimise unnecessary energy expenditure, and thus the altitude gains and losses along their movement trajectories. We expected roe deer to avoid risks, especially those linked to human disturbance. In this context, roe deer should select forested areas further away from roads during the day, and more open areas closer to roads during the night time [8, 40]. Moreover, when close to roads, roe deer should select areas with higher forest density [19] in order to seek protection from human disturbance, as shown for other ungulates [34, 51]. With regards to how road avoidance may vary among movement modes, we predicted that it should be strongest for resident individuals with relatively high site familiarity and weakest for dispersers that move longer distances across unknown landscapes, with intermediate levels for migratory individuals.

## Methods

### Animal relocation data and study areas

The data used for this study were obtained from the EURODEER database of the EUROMAMMALS initiative (www.euromammals.org), a collaborative science project that stores and manages spatial data of European mammals from across Europe. We initially considered data from 344 individuals fitted with GPS collars between 2004 and 2015, for a total of 2,264,497 locations. Intervals between consecutive locations spanned between 15 min and 12 h.

**Fig. 1:**
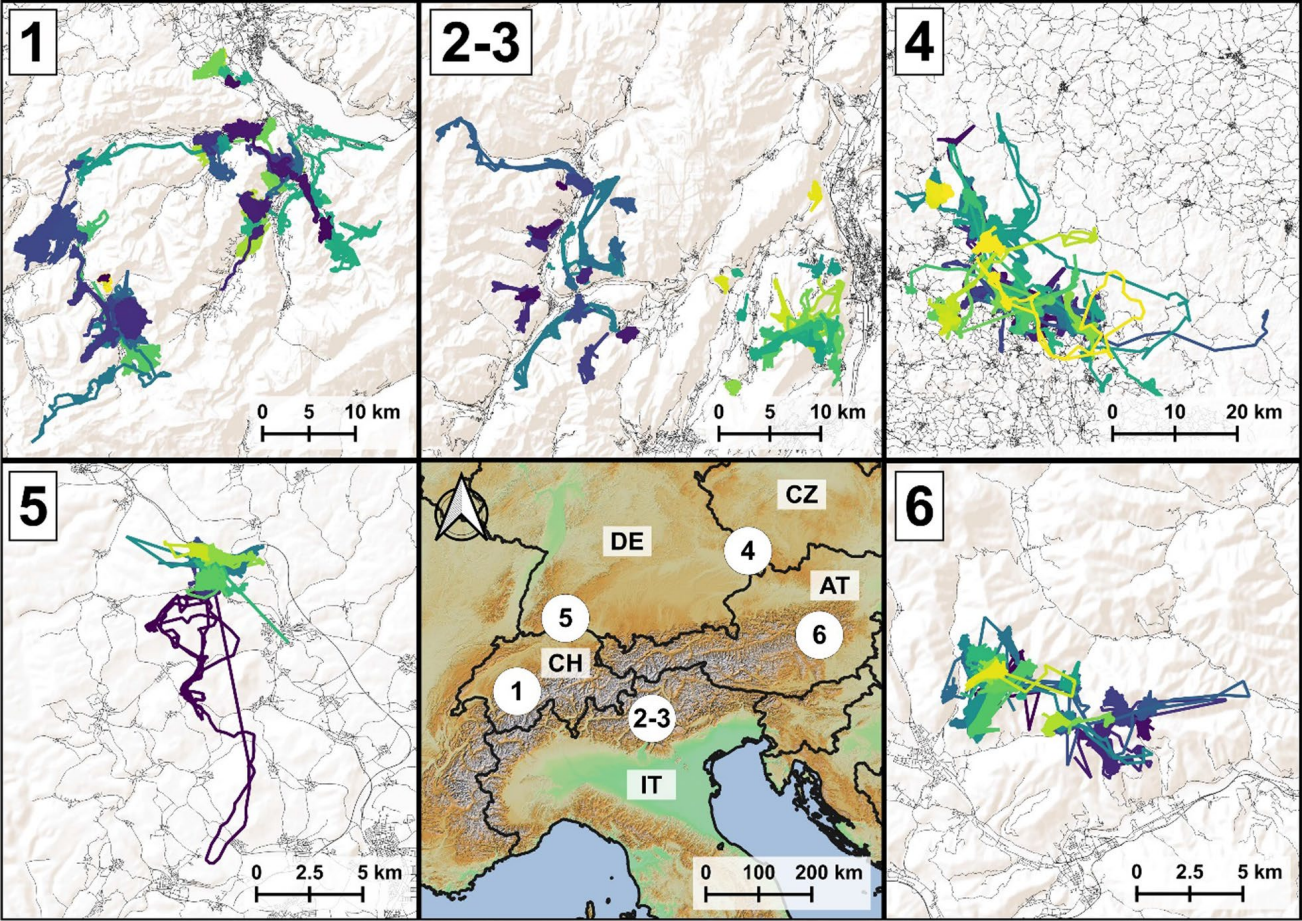
Map showing the location and key information of the 6 study areas (1 = Bernese, 2 = Rendena, 3 = Bondone, 4 = Bavaria, 5 = Hegau, 6 = Leoben). For each area, we show the trajectories of the GPS tracked roe deer, the road network and the digital elevation model

The collared individuals were from six different populations in mountainous or sub-mountainous areas in the Alps and central Europe (Fig. 1—Switzerland: Bernese Oberland (n = 74); Italy: Val Rendena (n = 27 individuals), Monte Bondone (n = 26 individuals); Germany: Bavarian Forest (n = 179 individuals), Hegau (n = 12 individuals); Austria: Leoben (n = 26 individuals)).

Despite being different in many aspects, the selected areas are characterised by a mountainous topography, the presence of natural or semi-natural areas, and a limited proportion of agricultural areas. Roads, human settlements and urban areas are mostly concentrated at the bottom of the valleys (see Additional file 1 for more information about study areas). Furthermore, individuals in these populations are known to perform all the movement modes considered in this study (i.e., home range movements, migration, dispersal).

### Movement data management and classification of movement patterns

From the initial dataset, we retained only the individuals with a fix interval of up to 5 h. Between January and March, movements are generally limited within the winter ranges. As such, we selected the GPS trajectories with at least 10 months of data from a start date between 1 and 31^st^ March to an end date between the 1st and 31st January of the following year. These dates were chosen based on Peters et al. [50] and Peters et al. [49] to ensure that we captured migration and dispersal events, and to maximise sample size. Each GPS trajectory was then truncated by discarding locations falling outside of our specified date window.

In order to classify movement patterns, we used the Net Squared Displacement method (NSD—[11]). The NSD is calculated as the squared geographical distance of each GPS location from a first, reference location. In this case, we ensured that the first location fell in an animal’s winter range. The NSD method fits several non-linear models to the observed NSD values, each representing a movement mode (i.e. resident, migrant, disperser). The best model, which classifies the movement pattern of each individual, is then selected based on the Akaike Information Criteria (AIC) [11]. The NSD analyses were conducted using the ‘migrateR’ package Spitz et al. [64]—parameters available in Additional file 1) in R 3.5.1 (R Development Core Team 2013). Animal trajectories were also spatially investigated using SeqScan, a spatio-temporal clustering algorithm available in the QGIS plugin ‘MigrO’ which has previously proved effective for the analysis of movement patterns from GPS trajectories [21, 22]. The use and comparison of multiple methods is recommended for a more accurate classification of movement patterns, especially in species like roe deer with a wide range of movement behaviours [12]. If the classification was ambiguous using the NSD method and SeqScan, the trajectory was discarded.

Because we were interested in comparing step selection among different movement modes, for migration and dispersal, we only retained the trajectory of the transience phase (i.e. the segment of trajectory during the period of migration and dispersal). To do this, we identified the start and end dates of these events from the NSD plot using the ‘*locator()*’ function in the ‘graphics’ R package and we used these dates to truncate the trajectories. As such, spring migration trajectories start from the time when an animal leaves its winter range to the time it settles in the summer range. Similarly, for dispersal trajectories, we considered only the movement between the two ranges, ignoring locations within the two ranges. Stopovers and multi-trip migrations (i.e. including multiple trips between seasonal ranges before settling—[13]) were included, as long as they occurred in the isolated segments. The trajectories of resident individuals were truncated using the median start and end times of migratory animals in order to evaluate behaviour during comparable periods. We report these values in the results section.

Once we classified each movement category and obtained the trajectories, we excluded all the individuals with a gap of more than 7 days of consecutive missing data. All trajectories were then re-sampled by retaining points at a fix interval of 3 to 5 h. We did not re-sample to a single fix interval because trajectories can only be re-sampled to a multiple of the original fix intervals, which in our case were 3, 4 and 5 h (i.e. the least common multiple would be 60 h, which is much larger than the scale we wanted to focus on). Following this process, we retained a total of 95 trajectories from 79 individuals, which represent the final dataset used in the model (Italy: Val Rendena (n = 10 individuals), Monte Bondone (n = 9); Germany: Bavarian Forest (n = 26), Hegau (n = 2); Switzerland: Bernese Oberland (n = 31); Austria: Leoben (n = 1)). The re-sampling of the trajectories was carried out using the R package ‘adehabitatLT’ [14].

### Integrated step selection analysis

In order to assess the impact of spatial variation in resources, topography, and roads on roe deer movement, we conducted an integrated step selection analysis (iSSA—[4]). iSSA jointly estimates resource selection and animal movement parameters (e.g. step length), by relaxing the implicit assumption that these are independent [4].

In iSSA, each observed animal step (i.e., movement between two consecutive GPS fixes) is compared to a set of random steps (i.e., that animal could have taken) using conditional logistic regression. In this study, we matched each observed step with 10 random steps, computed using distances sampled from a gamma distribution fitted to the empirical step length distribution and random turning angles, using the R package ‘amt’ [62] (see Additional file 1 for the step length and turning angle distributions used to generate random steps). Because our trajectories had different fix intervals ranging between 3 and 5 h, for each fix interval we generated random steps using the distribution of steps at the corresponding interval (e.g., random steps for an individual with a fix interval of 3 h were generated using the step distribution of individuals with a fix interval of 3 h) (see Additional file 1). The number of random steps was chosen based on the recommendations of Thurfjell et al. [67], to create a sufficiently large sample size while preventing excessively long computational processing times. To ensure that 10 random steps were enough, we performed a sensitivity analysis by running separate models using 1 to 10 random steps, and recorded model coefficients for each model (see Additional file 1).

We extracted environmental covariates at the end of each step, together with the number of road crossings along each step, the time of day (categorical: day, twilight, night—see Additional file 1 for details on how this was calculated) and step length (see Table 1 for the complete list of variables). All variables were scaled and centred, and screened for collinearity using the Pearson’s correlation coefficient with a threshold of |r| > 0.7 [25]. We performed an iSSA using mixed-effect conditional logistic regression to identify the main predictors of movement using the full set of covariates (see Table 2 for model terms), since we did not find any substantial collinearity between variables (see Additional file 1 for correlation matrix). To account for differences in habitat selection during different times of the day and when crossing roads [51], we added interactions between forest density and time of day, number of road crossings and time of day, and forest density and number of road crossings. To specifically test for differences in road avoidance among the three movement modes, we added the interaction between number of road crossings and the movement mode of each individual. To control for the fact that longer steps are statistically more likely to cross roads, we included an interaction between number of road crossings and the natural logarithm of step length [4]. To account for the fact that our trajectories had different fix intervals and to avoid biased model coefficients, we used the hourly step length, obtained by dividing each step length by the relative fix interval (Table 1, Additional file 1; see Additional file 1 also for model coefficients at different fix intervals). Finally, we controlled for potential differences in step length and number of road crossings for the two sexes. Nested random effects were included for individuals and populations to control for repeated measures. This decision was made to account for unequal number of steps for each individual and unequal numbers of individuals in each population. Models were fitted using the R package ‘coxme’ [65, 70]. We also attempted to account for variation among individuals by fitting random slopes using multiple approaches described by Muff et al. [45], Craiu et al. [20], and Therneau [65] unfortunately failing due to convergence issues (see Additional file 1 for the R code with the attempts to run these models and the section “Caveats and Potential Biases” in the Discussion).

**Table 1:** Variables used in the model. For each variable we report the resolution, a general description of the variable and the source from which the variable was obtained

Variable	Resolution	Description	Source
Elevation difference	25 m	Difference in altitude between start and end of a step	European Environment Agency (EEA) – EU Copernicus. European Digital Elevation Model (EU-DEM), version 1.1
Slope	25 m	Slope in degrees (0–90)	European Environment Agency (EEA) – EU Copernicus. European Digital Elevation Model (EU-DEM), version 1.1
Forest density	20 m	Percentage of area covered by trees (0–100%)	European Environment Agency, 2012
NDVI	250 m	Mean NDVI value (time series of 8 days composite)	Modis—Institute of Surveying, Remote Sensing and Land Information of the University of Natural Resources and Applied Life Sciences, Vienna (2004–2015)
Road crossings	–	Number of road crossings per step	Open street map. Only paved roads were considered
Time of day	–	Categorical (day, twilight, night) (see Additional file 1 for details on how it was calculated)	GPS collar data
Log(Step length)	–	Natural logarithm of hourly step length in meters (see Additional file 1)	GPS collar data
Movement mode	-	Categorical (Resident, migrant or disperser)	Analysis (see main text)
Sex	–	Categorical (Sex of the individual)	Collected at capture

**Table 2:** Terms used in the step selection function with the associated predictions

Model terms	Predictions
Forest Density + Forest density: Time of day	Selection of more forested areas during the day and more open areas during night time [8]
Slope + Slope^2^	Selection of flatter areas [31]. A quadratic term was added to evaluate selection of slopes with intermediate steepness
Elevation difference + Elevation difference^2^	Selection of steps with low elevation difference – i.e. low elevation gain/loss (i.e. around zero)
NDVI + NDVI^2^	Selection of areas with higher NDVI values. A quadratic term was added to evaluate selection of intermediate values, especially during migration [57]
Road crossings	General avoidance of road crossings [51]
Road crossings: Time of day	Road crossings should be avoided at all times of day, but particularly during the day and twilight, when traffic is more intense [40, 47]
Road crossings: Forest density	Road crossings should occur preferentially in more forested areas [19, 51]
Road crossings: Movement mode	Road avoidance should be strongest for resident individuals, intermediate for migrants and weakest for dispersers
log(Step length)	Statistical estimator of the parameters of the assumed step-length distribution [4]
log(Step length): Sex	Control to account for potentially different step lengths between sexes
Road crossings: log(Step length)	Control to account for the fact that longer steps are more likely to cross roads
Road crossings: Sex	Control to account for potentially different road avoidance between sexes

The importance of each explanatory variable in predicting habitat selection was assessed by removing one variable at a time from the full model, which included all predictors, and recording the change in AIC value for each sub-model (here called ΔAIC_REMOVED_). The most important variables were identified as those that, when removed, caused the highest increase in AIC (i.e. the variables with the highest ΔAIC_REMOVED_).

## Results

### Trajectories and movement patterns

Of the 95 retained trajectories, 25 were migratory events, 8 were dispersal events and 62 were extracted from resident individuals.

The median start date for migration was 6 April (min. 29 February; max. 3 May), while the median end date was 23 May (min. 25 March; max. 10 September). For dispersal, the median start date was 4 May (min. 28 February; max. 2 August) and the median end date was 7 June (min. 17 March; max. 20 August). For all resident animals, the start and end dates were set to 10 April and 27 May (i.e. the median start and end date for migration and dispersal), respectively. The median durations for migration, dispersal and resident movements were 61 days (min. 20; max. 192), 27 days (min. 16; max. 65) and 47 days (min. 47; max. 47; by definition), respectively.

### Integrated step selection analysis

In Table 3, we report the coefficients and confidence intervals of the full model, as well as the ΔAIC_REMOVED_ for each variable. The most important variables were forest density (ΔAIC_REMOVED_ = 1784), its interaction with time of day (ΔAIC_REMOVED_ = 1738), and number of road crossings (ΔAIC_REMOVED_ = 1076). Despite most of the other variables being significant, they were relatively less important for explaining step selection (Table 3).

**Table 3:** Summary of the full model showing model coefficients (β) with 95% confidence intervals, the standard deviation of the random effects, and the number of observations

Variable	Categories	β	95% C.I.	ΔAIC_REMOVED_
log(Step length)	–	− 0.407	[− 0.425, − 0.389]	4697
Forest density	–	0.347	[0.324, 0.371]	1784
Forest density : Time of day	Day (Ref.)	0.347	[0.324, 0.371]	1738
	Night	− 0.787	[− 0.825, − 0.749]	
	Twilight	− 0.423	[− 0.486, − 0.36]	
Road crossings	–	− 1.379	[− 1.505, − 1.252]	1076
Road crossings : log(Step length)	–	0.231	[0.205, 0.257]	284
log(Step length) : Sex	Female (Ref.)	− 0.407	[− 0.425, − 0.389]	142
	Male	0.143	[0.119, 0.168]	
Slope	–	0.107	[0.081, 0.133]	129
Slope^2^	–	− 0.076	[− 0.092, − 0.06]	98
Road crossings : Forest density	–	0.068	[0.046, 0.09]	44
Road crossings : Time of day	Day (Ref.)	− 1.379	[− 1.505, − 1.252]	29
	Night	0.110	[0.065, 0.156]	
	Twilight	0.047	[− 0.034, 0.129]	
Elevation difference^2^	–	− 0.014	[− 0.02, − 0.007]	28
Elevation difference	–	0.003	[− 0.015, 0.021]	26
NDVI	–	0.056	[0.005, 0.107]	24
Road crossings : Sex	Female (Ref.)	− 1.379	[− 1.505, − 1.252]	16
	Male	− 0.066	[− 0.111, − 0.02]	
NDVI^2^	–	− 0.023	[− 0.047, 0.002]	11
Road crossings : Movement mode	Resident (Ref.)	− 1.379	[− 1.505, − 1.252]	7
	Disperser	0.010	[− 0.061, 0.081]	
	Migrant	0.030	[− 0.023, 0.084]	

Random effects	Standard deviation			
Individual animals (N=79)	0.041			
Populations (N=6)	0.020			
Number of events: 26892 observed + 268920 random steps				

“Ref.” indicates the category of reference for categorical variables. For each variable, we also report the ΔAIC_REMOVED_, i.e., the difference in AIC between the full model and the full model without that variable. Hence, the variables with the highest ΔAIC_REMOVED_ are the most important because, when removed, they caused the highest increase in AIC. Variables are listed in order of importance. The AIC of the full model including all variables was 129,748.

**Fig. 2:**
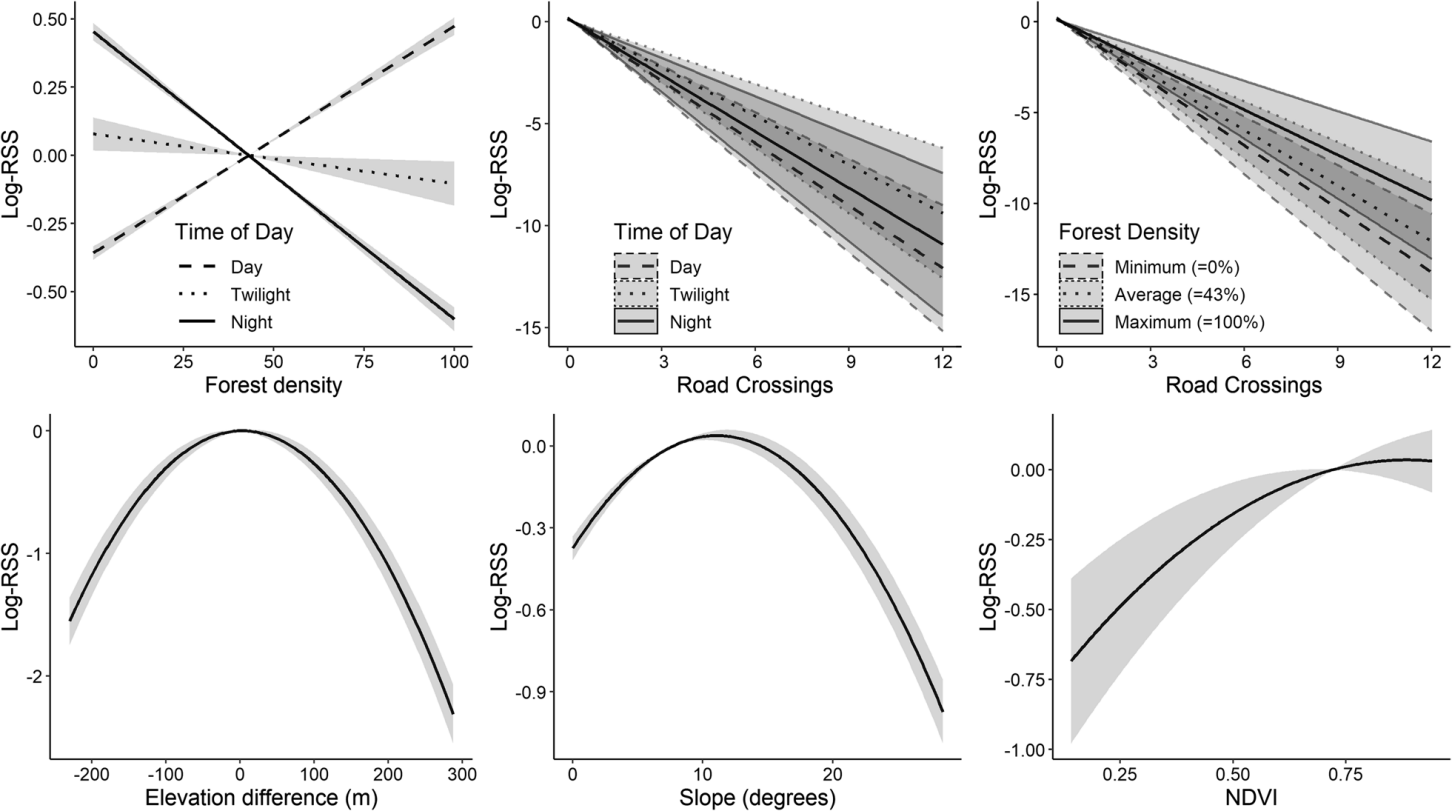
Response curves calculated using the fixed effect coefficients of the step selection function and showing the Relative Selection Strength (Log-RSS) for the most important model predictors with 95% confidence intervals (shaded areas) [3]. The range of values of the predictors on the x-axis corresponds to observed ranges, thus meaning that we did not project the predictions to unobserved or unrealistic values

In general, roe deer tended to select steps with higher forest density during the day, but with more open habitat during night time (day: β = 0.35; 95% CI [0.32, 0.37], night compared to day: β = − 0.79; 95% CI [− 0.83, − 0.75]—Table 2, Fig. 2). Roads were strongly avoided at all times of the day (β = − 1.38; 95% CI [− 1.51, − 1.25]), but were crossed more frequently in areas with higher forest density (β = 0.07; 95% CI [0.05, 0.09]). The number of road crossings increased with step length (β = 0.23; 95% CI [0.21, 0.26]). Furthermore, males tended to have longer steps than females (females: β = − 0.407; 95% CI [− 0.43, − 0.39]; males vs females: β = 0.143; 95% CI [0.119, 0.168]).

On average, roe deer tended to select areas with slopes of intermediate steepness, at around 10° (β = − 0.08; 95% CI [− 0.09, 0.06]), and minimised altitude gains or losses along their steps, as shown by the negative coefficient for the squared elevation difference term (β = − 0.01; 95% CI [− 0.02, − 0.01]). NDVI was the penultimate variable in the model in terms of importance (ΔAIC_REMOVED_ = 24), despite having a positive and significant coefficient (β = 0.06; 95% CI [0.01, 0.11]). The interaction between movement mode and number of road crossings was not significant (dispersers compared to residents: β = 0.01; 95% CI [− 0.06, 0.08], migrants compared to residents: β = 0.03; 95% CI [− 0.02, 0.08]) and was the least important variable in our model (ΔAIC_REMOVED_ = 7).

## Discussion

Using an integrated step selection approach, we investigated habitat selection of roe deer during various movement modes across six populations in mountainous regions with a significant human footprint. We showed that roe deer mainly selected forested areas, minimised elevation differences along their movement trajectories, and consistently minimised the number of roads crossed during migration, dispersal, and home range movements.

As expected, we found that higher forest densities were selected during the day, while less forested areas were visited more frequently at night, in accordance with other studies investigating roe deer habitat selection [8, 23]. Roads were strongly avoided at all times of the day, and crossings tended to be more common during longer movements. Roads have been shown to limit movement and space use in many animal taxa, including large carnivores [18], small mammals [33], reptiles [61], amphibians [15], and even fish [48]. In ungulates, road crossings were avoided also by elk (*Cervus canadensis*), pronghorn (*Antilocapra americana*) and moose in North America [7, 51, 60], and reindeer in Norway [6]. Studies on roe deer had previously suggested that they avoid roads at the home range scale, although focusing on the distance to the nearest road [19], rather than actual crossings. We found that when animals did cross roads, they also tended to end their steps in more forested areas, potentially to seek protection and reduce risk of exposure to predators and disturbance, as shown for elk in North America [51]. Coulon et al. [19] and Bonnot et al. [8] also showed that roe deer selected forested areas more strongly when in proximity of roads.

In general, roe deer selected slopes of intermediate steepness (~ 10°) as also found by Ranc et al. [52]. However, this differed from other studies arguing that roe deer should select flatter areas to minimise energy expenditure [31]. Our result may be due to the fact that, in human-dominated mountainous areas like those considered here, urbanisation and human activities are usually concentrated in the lower and flatter parts of the valley. Nonetheless, minimisation of energy expenditure is likely the reason why we found that roe deer also tended to minimise elevation gained or lost along their movements. Moreover, in summer, mountain ungulates tend to follow the elevation green up of the vegetation (‘surfing the green wave’, e.g. Aikens et al. [1]. Hence, excessively large altitudinal displacements may result in a spatial mismatch between the location of the animal and the distribution of optimal resources [1]. In the French Alps, larger displacements towards high elevations were observed in correspondence of higher primary productivity, where more abundant resources could compensate for higher movement costs [31]. In our case, NDVI had only a weak positive effect on roe deer step selection. This may be due to the fact that, in human-dominated landscapes, the effects of anthropogenic disturbance on ungulate behaviour can exceed those of natural processes [16]. It is therefore possible that roe deer are forced to select sub-optimal resources in order to avoid roads and other sources of human disturbance [16]. Nonetheless, it is also possible that the resolution of the NDVI variable considered in this study (8-day average at 250 m) may be too coarse to capture its effect on roe deer step selection (average step length ≈ 159 m in 3 h). In accordance with this, Aikens et al. [2] showed that roe deer do not follow the plant green up as closely as other species, but select the most productive habitats at a finer scale [43].

We did not find any evidence that road avoidance varied across migration, dispersal or home range movements. In other words, although roe deer may be less familiar with the landscape, and may be forced to frequently cross roads during migration and dispersal [5], their level of road avoidance remained high and consistent with that expressed during home range movements. Roads can therefore pose a significant barrier for migrating roe deer to reach seasonal ranges and potentially access better resources [50, 63], and for dispersing roe deer to establish in a novel range, thus connecting spatially distinct populations [26]. In a partially migratory species, this may thus constrain migratory movements and reduce the relative proportion of migrants in high-road density areas. However, road crossings inevitably still occur, and resulting collisions with vehicles represent an important cause of mortality for roe deer in Europe, especially in relation to migration and dispersal events [55]. Additionally, even with regards to resident movements, the high level of road avoidance suggests a general constraint of human infrastructure on roe deer mobility. Future studies should focus on investigating fine scale behavioural adjustments to such constraints. For example, roads, as a source of risk, might be kept at the periphery of the home range (Seigle-Ferrand et al. unpubl.). This hypothesis is supported by recent work on the role of familiarity in shaping resource use [52, 54] and risk avoidance, whereby roe deer are more likely to constrain their movements to familiar areas, even within an established home range, where they experience lower mortality [32].

Further research is also needed to evaluate the impact and consequences of human disturbance for dispersal and metapopulation functioning. We showed that road avoidance behaviour did not vary between resident (n = 62) and migratory (n = 25) individuals. However, due to low data availability, we could not draw strong conclusions for dispersing individuals (n = 8). Another priority for research is to integrate other behavioural processes among the drivers of movement. For example, it has been shown that memory plays an important role in ungulate movement and space use [10]. However, most studies integrating memory in habitat selection analysis have focused on space use within the home range, with a few exceptions [10, 44, 52]. Finally, future research should focus on relating movement and habitat selection to energetics and demography to quantify their costs and benefits for individual performance and ultimately population dynamics.

### Caveats and potential biases

In our model, we included random intercepts for individuals and populations to account for repeated measures. In order to obtain more accurate estimates and account for variation among individuals [45], we also attempted to include random slopes using multiple recently-developed methods, unfortunately without success. First, we tried using ‘coxme()’ [65] and the approaches proposed by Muff et al. [45]. These approaches worked well for simpler formulations of our model, providing very similar outputs as obtained without random slopes, however they failed to provide outputs as soon as the model became more complex (e.g., inclusion of interactions and nested random slopes) due to convergence issues. Indeed, these models can be challenging to fit [28, 45]. We also tried to use the two-step approach described in Craiu et al. [20], but this was not applicable as the values of several variables in our model remained constant within all strata of at least one cluster. This is the main limitation of the two-step approach [20, 28, 45]. The R code used to run all the above-mentioned models is provided in Additional file 1.

Including mixed effects in conditional logistic regressions remains challenging and is a developing topic in iSSA [28]. The use of random slopes has been recommended to fully account for inter-individual heterogeneity [45]. Indeed, random-intercept-only models like the one presented here cannot account for among-individual variation in the regression slopes. Nonetheless, while we think that individual variation is important, the main aim of this study was to investigate how roads and other environmental factors can affect roe deer movement at the population level. According to recent findings, adding random slopes to our model could have produced more accurate estimates and allowed for more variance around random intercepts [45]. However, iSSAs including random slopes have also been shown to produce biased parameters when movement characteristics were included in the model (e.g. step length) [28, 45].

In conclusion, we are aware that including random slopes would have better accounted for uncertainty around our coefficients and possibly resulted in different estimates of effect sizes [28]. However, given the computational challenges and the scarcity of research on mixed-effect iSSAs, we could not implement any of the currently available approaches in practice. Finally, considering the coherence of our results with the vast literature on roe deer ecology, and the consistency of outputs between the different approaches when using simpler model formulations with and without random slopes, it is unlikely that adding random slopes to the full model would have significantly changed our key findings.

## Conclusions

This study showed that roads can represent a major constraint to movement across populations and movement modes, even for a species with relatively low movement propensity, and can therefore limit ecological connectivity at different scales [39]. This is particularly relevant in mountainous areas, where roads are generally concentrated along valley bottoms, and high mountains can also represent barriers to individual movement and population spatial distribution. In the future, a spatially explicit model integrating other wide-ranging Alpine species (e.g. red deer, chamois, lynx, bear and wolf), could help predict the areas where roads have the strongest impact on functional connectivity, thus providing important guidance for targeted policy decisions and management interventions at the landscape scale.

## Supplementary Information

Additional file 1. **Chapter 1**: Study area summary statistics. **Chapter 2**: MigrateR parameters. **Chapter 3**: Generation of random steps and use of step length as a variable in the model. **Chapter 4**: Sensitivity analysis for number of available steps. **Chapter 5**: Definition of the variable “Time of day”. **Chapter 6**: Collinearity of model predictors. **Chapter 7**: Model coefficients for steps at 3- and 5-hour fix intervals. **Chapter 8**: R code used to fit random slopes models.

## Data Availability

All raw data used in this article are stored in the EURODEER spatial database hosted by the Fondazione Edmund Mach (https://euromammals.org) and can be accessed upon login.

## Abbreviations

NSD: Net squared displacement
iSSA: Integrated step selection analysis
NDVI: Normalised difference vegetation index
AIC: Akaike Information Criteria
