# Supplementary material for: Roads constrain movement across behavioural processes in a partially migratory ungulate
Source: Mov Ecol. 2021 Nov 13;9:57. doi: 10.1186/s40462-021-00292-4 (PMC8590235; doi:10.1186/s40462-021-00292-4)
Supplement: Supplementary file 1 — Additional file 1. Chapter 1: Study area summary statistics. Chapter 2: MigrateR parameters. Chapter 3: Generation of random steps and use of step length as a variable in the model. Chapter 4: Sensitivity analysis for number of available steps. Chapter 5: Definition of the variable “Time of day”. Chapter 6: Collinearity of model predictors. Chapter 7: Model coefficients for steps at 3- and 5-hour fix intervals. Chapter 8: R code used to fit random slopes models. [file 40462_2021_292_MOESM1_ESM.docx]

Additional Files for:

Roads constrain movement across behavioural processes in a partially migratory ungulate

In: Movement Ecology

Authors:

Gioele Passoni, Tim Coulson, Nathan Ranc, Andrea Corradini, A.J.M. Hewison, Simone Ciuti, Benedikt Gehr, Marco Heurich, Falko Brieger, Robin Sandfort, Atle Mysterud, Niko Balkenhol, Francesca Cagnacci.

Contents

[Additional File 1. Study area summary statistics 2](#_Toc84605609)

[Additional File 2. MigrateR parameters 3](#_Toc84605610)

[Additional File 3. Generation of random steps and use of step length as a variable in the model. 4](#_Toc84605611)

[Additional File 4. Sensitivity analysis for number of available steps 7](#_Toc84605612)

[Additional File 5. Definition of the variable “Time of day” 8](#_Toc84605613)

[Additional File 6. Collinearity of model predictors 9](#_Toc84605614)

[Additional File 7. Model coefficients for steps at 3- and 5-hour fix intervals. 10](#_Toc84605615)

[Additional File 8. R code used to fit random slopes models 11](#_Toc84605616)

[1.Coxme (Therneau, 2015) 11](#_Toc84605617)

[2.Two-step clogit (Craiu et al., 2011) 11](#_Toc84605618)

[3.INLA (Muff et al., 2020) 12](#_Toc84605619)

[4.GlmmTMB (Muff et al., 2020) 13](#_Toc84605620)

# Additional File 1. Study area summary statistics

Table 1 Summary statistics for the six study areas including environmental variables (mean [min-max]) and final number of individuals, trajectories and steps used in the model. *The fix interval in Leoben is actually 3 hours 15 minutes.

| **Study Area** | **Area (km^2^)** | **Elevation (m)** | **Forest density (%)** | **Slope (degrees)** | **Road density (km/km^2^)** | **No. of trajectories** | **No. of individuals** | **No. of steps per fix interval** | | |
| --- | --- | --- | --- | --- | --- | --- | --- | --- | --- | --- |
|  |  |  |  |  |  |  |  | **3h** | **4h** | **5h** |
| Bondone | 194 | 872  [183-2125] | 63  [0-100] | 11  [0-34] | 2.36 | 9 | 9 | 0 | 1930 | 0 |
| Bavaria | 1629 | 888  [417-1441] | 62  [0-100] | 4  [0-24] | 1.12 | 31 | 26 | 515 | 546 | 5788 |
| Leoben | 86 | 1025  [568-1639] | 72  [0-100] | 10  [0-20] | 0.64 | 1 | 1 | 139* | 0 | 0 |
| Rendena | 357 | 1465  [461-3135] | 59  [0-100] | 13  [0-34] | 1.26 | 10 | 10 | 3376 | 0 | 0 |
| Bernese | 1047 | 1474  [558-3002] | 33  [0-98] | 11  [0-34] | 1.30 | 42 | 31 | 14465 | 0 | 0 |
| Hegau | 71 | 644  [455-874] | 21  [0-94] | 4  [0-17] | 2.08 | 2 | 2 | 133 | 0 | 0 |
| **Total** |  |  |  |  |  | **95** | **79** | **18489** | **2476** | **5788** |

# Additional File 2. MigrateR parameters

Table 2 List of migrateR parameters used for the NSD analysis .

| **Parameter** | **Description** | **Value** | **Unit** |
| --- | --- | --- | --- |
| l.d | NSD separating the first and second range (lower constraint) | 5 | Km^2^ |
| s.d | NSD separating the first and second range (starting value) | 100 | Km^2^ |
| l.r | Duration of occupancy on second range (lower constraint) | 60 | Days |
| u.r | Duration of occupancy on second range (starting value) | 270 | Days |
| l.g | Mean NSD of resident range (lower constraint) | 0.07 | Km^2^ |
| s.g | Mean NSD of resident range (starting value) | 3 | Km^2^ |
| u.g | Mean NSD of resident range (upper constraint) | 5 | Km^2^ |
| l.p | Time to complete 1/2 to 3/4 of departing movement (lower constraint) | 1 | Days |
| s.p | Time to complete 1/2 to 3/4 of departing movement (starting value) | 1 | Days |
| s.p2 | Time to complete 1/2 to 3/4 of returning movement (starting value) | 1 | Days |
| u.p2 | Time to complete 1/2 to 3/4 of returning movement (upper constraint) | 1 | Days |
| l.z | Difference in distance separating second and third range (lower constraint) | 0.03 | % |

# Additional File 3. Generation of random steps and use of step length as a variable in the model.

Random steps were generated using the R package “amt” (Signer, 2018). Because our GPS data for two of the six populations had different fix intervals between 3 and 5 hours, for each fix interval we sampled random steps from its respective distribution (e.g. for individuals with 3 hours fix interval, we sampled the random steps from a distribution drawn using observed steps at 3 hours fix interval). This can be seen in figure 1A, which shows that individuals with larger fix intervals have longer generated steps on average (slope = 79.6; 95% C.I. = [59.3; 99.9]).

To account for these differences in our model, we normalised the step length in figure 1A by dividing it by the fix interval, thereby obtaining the hourly step length (m/h). As can be seen in figure 1B, the newly created variable (i.e. step length per hour) does not significantly change with fix interval (slope = 2.1; 95% C.I. = [-2.8; 7.0]). In our model, we therefore used the normalised variable ‘step length per hour’ to avoid a biased estimation of the model coefficients.

In table 3, we also report raw summary statistics. In figure 2, we show the distributions of used and available steps as a function of all habitat covariates at 3- and 5-hour fix intervals. Finally, in table 4, we report the parameters of the step length and turn angle distributions used to generate random steps.

Figure 1 Plots showing the mean step length in metres (A) and the mean hourly step length in metres per hour (B) of generated steps as a function of fix interval. Each point represents the mean value of each individual animal. In each graph, we report the slope coefficient and 95% confidence intervals of a linear model fitted through the data (grey line and shadow).


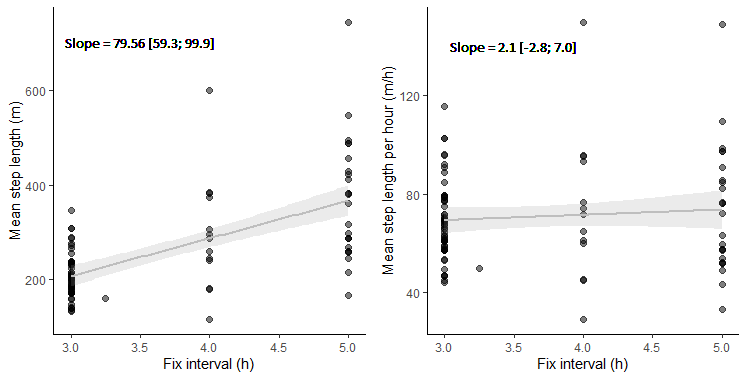


A

B

Table 3 Table showing summary statistics for step length (m) and hourly step length (m/h) of the generated steps for each fix interval.

| **Fix interval (hh:mm)** | **No. of individuals** | **Step length (m)** | | | | **Hourly step length (m/h)** | | | |
| --- | --- | --- | --- | --- | --- | --- | --- | --- | --- |
|  |  | **Min** | **Mean** | **Median** | **Max** | **Min** | **Mean** | **Median** | **Max** |
| **03:00** | 56 | 0 | 208 | 164 | 2883 | 0 | 60 | 44 | 1306 |
| **03:15** | 1 | 1 | 162 | 136 | 838 | 0 | 50 | 42 | 258 |
| **04:00** | 13 | 0 | 296 | 211 | 3301 | 0 | 74 | 53 | 825 |
| **05:00** | 25 | 0 | 365 | 262 | 5761 | 0 | 73 | 52 | 1152 |


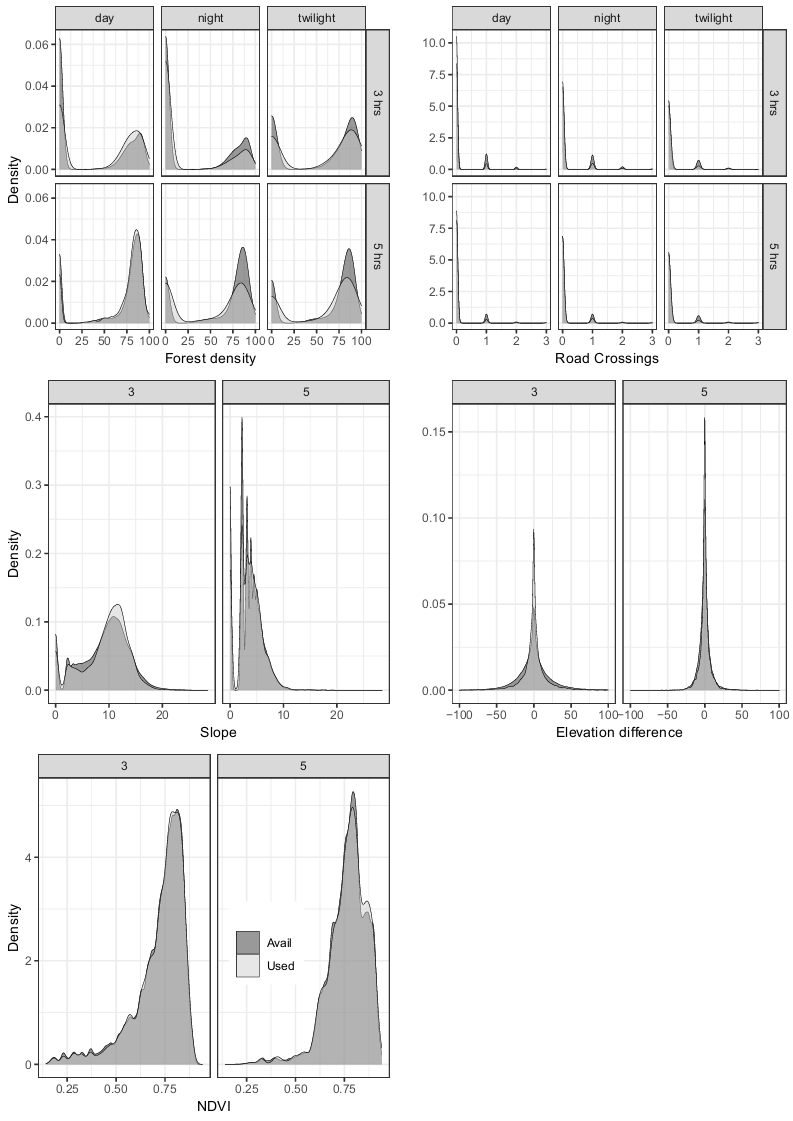


Figure 2 Distributions of used and available steps as a function of all habitat covariates at 3- and 5-hour fix intervals

Table 4 Parameters of the step length and turn angle distributions used to generate random steps.

|  |  |  | **Fix interval (hours)** | | | |
| --- | --- | --- | --- | --- | --- | --- |
| **Metric** | **Distribution** | **Parameter** | **3** | **3.25** | **4** | **5** |
| Step length | Gamma | Scale | 0.022 | 0.038 | 0.018 | 0.019 |
| Step length | Gamma | Shape | 1.547 | 1.901 | 1.384 | 1.341 |
| Turning angle | Von Mises | Kappa | 0.006 | 0.038 | 0.022 | 0.011 |

# Additional File 4. Sensitivity analysis for number of available steps

To ensure that 10 random steps were enough for the estimation of the iSSF, we performed a sensitivity analysis by running separate models using 1 to 10 random steps, recording model coefficients for each model.
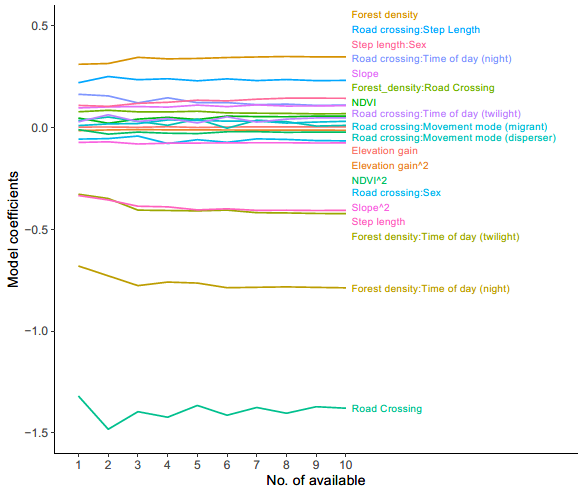


Figure 3 Sensitivity analysis to determine the number of available steps in the iSSF. The figure shows the change in model coefficient for each variable as a function of the number of available steps used in each model.

# Additional File 5. Definition of the variable “Time of day”

In our model, Time of day is a categorical variable with three levels: day, night and twilight. This categorisation was performed taking into account the position of the sun using the ‘getSunlightTimes()’ function in the ‘suncalc’ package in R.

The three levels are defined as follows:

- **Day**: starts at the end of sunrise (i.e. when the bottom edge of the sun touches the horizon) and ends at the beginning of sunset (i.e. when the bottom edge of the sun touches the horizon);
- **Night**: starts at dusk (i.e. start of evening nautical twilight and end of civil twilight with sun at 6˚) and ends at dawn (i.e. end of morning nautical twilight and start of morning civil twilight with sun at 6˚);
- **Twilight**:
  - starts at dawn (i.e. end of morning nautical twilight and start of morning civil twilight with sun at 6˚) and ends at the end of sunrise (i.e. when the bottom edge of the sun touches the horizon), and;
  - starts at beginning of sunset (i.e. when the bottom edge of the sun touches the horizon) and ends at dusk (i.e. start of evening nautical twilight and end of civil twilight with sun at 6˚).

The categorisation is summarised in the table below, together with the number of steps included in each time of day category.

|  | **Sunlight times** | **Definition** | **Time of day category in this paper** | **Number of steps per fix interval** | | |  |
| --- | --- | --- | --- | --- | --- | --- | --- |
|  |  |  |  | 3h | 4h | 5h | |
| **Time** | Sunrise end | Bottom edge of the sun touches the horizon |  |  |  |  | |
|  |  |  | Day | 11173* | 1282 | 3262 | |
|  | Sunset start | Bottom edge of the sun touches the horizon |  |  |  |  | |
|  |  |  | Twilight | 958* | 418 | 823 | |
|  | Dusk | Start of evening nautical twilight and end of civil twilight |  |  |  |  | |
|  |  |  | Night | 6497* | 776 | 1703 | |
|  | Dawn | End of morning nautical twilight and start of morning civil twilight |  |  |  |  | |
|  |  |  | Twilight | as above | | | |
|  | Sunrise end | Bottom edge of the sun touches the horizon |  |  |  |  | |

*of these 79, 21 and 39 steps (for day, twilight and night respectively) had a fix interval of 3 hours 15 minutes.

# Additional File 6. Collinearity of model predictors


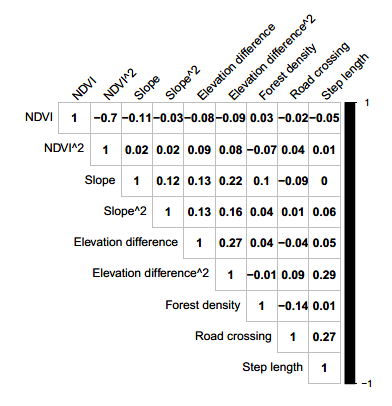
We screened for potential collinearity among model predictors using the Pearson’s correlation coefficient (threshold of |r|> 0.7 - Dormann et al. (2013)) (Figure below)

Figure 4 Pearson's correlation coefficients (R) for model predictors.

Figure 5 Pearson's correlation coefficients (R) for model predictors.

# Additional File 7. Model coefficients for steps at 3- and 5-hour fix intervals.


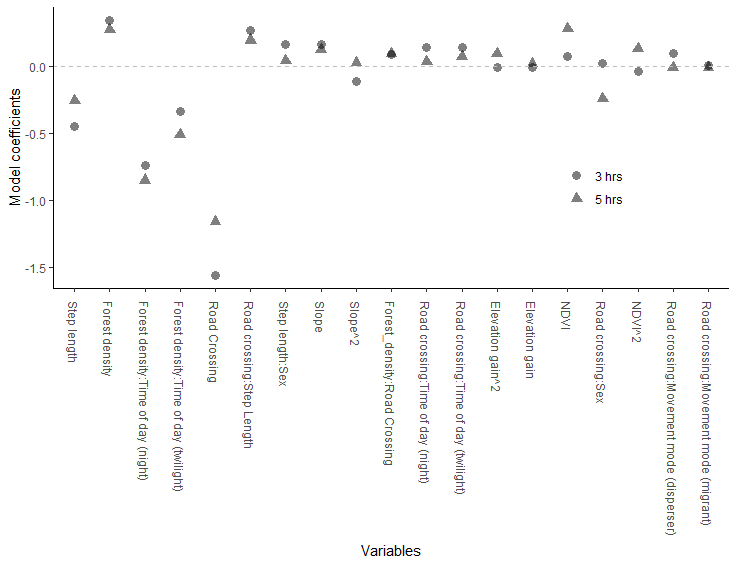


Figure 6 Model coefficients for steps at 3- and 5-hour fix intervals.

# Additional File 8. R code used to fit random slopes models

We report the R code that we created to attempt to fit the random slopes models using four different approaches, together with the reason why they did not work. For some approaches, we did not include interactions between predictors as random slopes as the model did not converge even with simpler formulations.

## 1.Coxme (Therneau, 2015)

coxme_rdm <- coxme(formula = Surv(time = ones, event = use_avail) ~

ndvi + I(ndvi^2) + slope + I(slope^2) + forest_density + elevation_diff +

I(elevation_diff^2) + forest_density:timeofday + road_cross + road_cross:sex +

step_length + step_length:sex + road_cross:step_length + road_cross:forest_density +

road_cross:timeofday + road_cross:move_mode + strata(step_id) +

(ndvi | study_area) + (ndvi | animal_id) +

(I(ndvi^2) | study_area) + (I(ndvi^2) | animal_id) +

(slope | study_area) + (slope | animal_id) +

(I(slope^2) | study_area) + (I(slope^2) | animal_id) +

(forest_density | study_area) + (forest_density | animal_id) +

(elevation_diff | study_area) + (elevation_diff | animal_id) +

(I(elevation_diff^2) | study_area) + (I(elevation_diff^2) | animal_id) +

(road_cross | study_area) + (road_cross | animal_id) +

(step_length | study_area) + (step_length | animal_id),

data = dat, na.action = "na.fail")

*We interrupted the model after 24 hours of non-convergence.*

## 2.Two-step clogit (Craiu et al., 2011)

twostep_rdm <- Ts.estim(formula = use_avail ~

ndvi + I(ndvi^2) + slope + I(slope^2) + forest_density +

elevation_diff + I(elevation_diff^2) + forest_density:timeofday +

road_cross + road_cross:sex + step_length + step_length:sex +

road_cross:step_length + road_cross:forest_density + road_cross:timeofday +

road_cross:move_mode + strata(step_id) + cluster(animal_id),

data = dat,

random = ~ ndvi + I(ndvi^2) + slope + I(slope^2) + forest_density +

elevation_diff + I(elevation_diff^2) + forest_density:timeofday +

road_cross + road_cross:sex + step_length + step_length:sex +

road_cross:step_length + road_cross:forest_density +

road_cross:timeofday + road_cross:move_mode,

all.m.1=F, D="UN(1)")

*The model could not be fitted because the value of several variables remains constant within all strata of at least one cluster.*

## 3.INLA (Muff et al., 2020)

Zlt <- as(model.matrix(~ 0 + study_area:animal_id, data = dat), "Matrix")

inla_rdm_formula <- use_avail ~ -1 + forest_density + road_cross + ndvi + I(ndvi^2) +

slope + I(slope^2) + step_length + forest_density:timeofday + elevation_diff +

I(elevation_diff^2) + road_cross:timeofday + road_cross:forest_density + road_cross:move_mode +

f(step_id, model="iid",hyper=list(theta = list(initial=log(1e-6),fixed=T))) +

f(IDi1,road_cross, model="z", Z=Zlt, hyper=list(theta=list(initial=log(1),fixed=F,prior="pc.prec",param=c(3,0.05)))) +

f(IDi2, forest_density, model="z", Z=Zlt, hyper=list(theta=list(initial=log(1),fixed=F,prior="pc.prec",param=c(3,0.05)))) +

f(IDi3, ndvi, model="z", Z=Zlt, hyper=list(theta=list(initial=log(1),fixed=F,prior="pc.prec",param=c(3,0.05)))) +

f(IDi4, I(ndvi^2), model="z", Z=Zlt, hyper=list(theta=list(initial=log(1),fixed=F,prior="pc.prec",param=c(3,0.05)))) +

f(IDi5, slope, model="z", Z=Zlt, hyper=list(theta=list(initial=log(1),fixed=F,prior="pc.prec",param=c(3,0.05)))) +

f(IDi6, I(slope^2), model="z", Z=Zlt, hyper=list(theta=list(initial=log(1),fixed=F,prior="pc.prec",param=c(3,0.05)))) +

f(IDi7, elevation_diff, model="z", Z=Zlt, hyper=list(theta=list(initial=log(1),fixed=F,prior="pc.prec",param=c(3,0.05)))) +

f(IDi8, I(elevation_diff^2), model="z", Z=Zlt, hyper=list(theta=list(initial=log(1),fixed=F,prior="pc.prec",param=c(3,0.05))))

inla_rdm <- inla(inla_rdm_formula, family ="Poisson", data=dat,

control.fixed = list(mean = mean.beta, prec = list(default = prec.beta)), verbose=TRUE)

*The model did not converge and stopped running after more than 5 hours.*

## 4.GlmmTMB (Muff et al., 2020)

tmb_struc_rdm = glmmTMB(use_avail ~

ndvi + I(ndvi^2) + slope + I(slope^2) + forest_density + elevation_diff +

I(elevation_diff^2) + forest_density:timeofday + road_cross + road_cross:sex +

step_length + step_length:sex + road_cross:step_length +

road_cross:forest_density + road_cross:timeofday + road_cross:move_mode +

(1 | step_id) + (0 + ndvi | study_area/animal_id) +

(0 + I(ndvi^2) | study_area/animal_id) + (0 + slope | study_area/animal_id) +

(0 + I(slope^2) | study_area/animal_id) +

(0 + forest_density | study_area/animal_id) +

(0 + elevation_diff | study_area/animal_id) +

(0 + I(elevation_diff^2) | study_area/animal_id) +

(0 + forest_density:timeofday | study_area/animal_id) +

(0 + road_cross | study_area/animal_id) +

(0 + road_cross:sex | study_area/animal_id) +

(0 + step_length | study_area/animal_id) +

(0 + step_length:sex | study_area/animal_id) +

(0 + road_cross:step_length | study_area/animal_id) +

(0 + road_cross:forest_density | study_area/animal_id) +

(0 + road_cross:timeofday | study_area/animal_id) +

(0 + road_cross:move_mode | study_area/animal_id),

family=poisson, data=dat, doFit = FALSE)

tmb_struc_rdm$parameters$theta[1] = log(1e3)

tmb_struc_rdm$mapArg = list(theta=factor(c(NA,1:(length(tmb_struc_rdm$parameters$theta)-1))))

glmmTMB_rdm <- glmmTMB:::fitTMB(tmb_struc_rdm)

*The model did not converge and stopped running after 10’.*

**References:**

CRAIU, R. V., DUCHESNE, T., FORTIN, D. & BAILLARGEON, S. 2011. Conditional Logistic Regression With Longitudinal Follow-up and Individual-Level Random Coefficients: A Stable and Efficient Two-Step Estimation Method. *Journal of Computational and Graphical Statistics,* 20**,** 767-784.

DORMANN, C. F., ELITH, J., BACHER, S., BUCHMANN, C., CARL, G., CARRÉ, G., MARQUÉZ, J. R. G., GRUBER, B., LAFOURCADE, B., LEITÃO, P. J., MÜNKEMÜLLER, T., MCCLEAN, C., OSBORNE, P. E., REINEKING, B., SCHRÖDER, B., SKIDMORE, A. K., ZURELL, D. & LAUTENBACH, S. 2013. Collinearity: a review of methods to deal with it and a simulation study evaluating their performance. *Ecography,* 36**,** 27-46.

MUFF, S., SIGNER, J. & FIEBERG, J. 2020. Accounting for individual-specific variation in habitat-selection studies: Efficient estimation of mixed-effects models using Bayesian or frequentist computation. *J Anim Ecol,* 89**,** 80-92.

SIGNER, J. 2018. amt: Animal Movement Tools. *R package version 4*.

THERNEAU, T. M. 2015. Package ‘coxme’: Mixed effects cox models. *R package version 2*.
